# Control of Ge island coalescence for the formation of nanowires on silicon†

**DOI:** 10.1039/d3nh00573a

**Published:** 2024-02-12

**Authors:** Santhanu Panikar Ramanandan, Joel Reñé Sapera, Alban Morelle, Sara Martí-Sánchez, Alok Rudra, Jordi Arbiol, Vladimir G. Dubrovskii, Anna Fontcuberta i Morral

**Affiliations:** a Laboratory of Semiconductor Materials, Institute of Materials, Ecole Polytechnique Fédérale de Lausanne EPFL Lausanne 1015 Switzerland anna.fontcuberta-morral@epfl.ch; b Solid State Laboratory, ETH Zurich 8093 Zurich Switzerland; c Catalan Institute of Nanoscience and Nanotechnology (ICN2), CSIC and BIST, Campus UAB, Bellaterra Barcelona Catalonia Spain; d Institute of Physics, Faculty of Basic Sciences, Ecole Polytechnique Fédérale de Lausanne EPFL Lausanne 1015 Switzerland; e ICREA, Pg. Lluís Companys 23 08010 Barcelona Catalonia Spain; f Faculty of Physics, St. Petersburg State University, Universitetskaya Embankment 13B 199034 St. Petersburg Russia; g Center for Quantum Science and Engineering, École Polytechnique Fédérale de Lausanne (EPFL) CH-1015 Lausanne Switzerland

## Abstract

Germanium nanowires could be the building blocks of hole-spin qubit quantum computers. Selective area epitaxy enables the direct integration of Ge nanowires on a silicon chip while controlling the device design, density, and scalability. For this to become a reality, it is essential to understand and control the initial stages of the epitaxy process. In this work, we highlight the importance of surface treatment in the reactor prior to growth to achieve high crystal quality and connected Ge nanowire structures. In particular, we demonstrate that exposure to AsH_3_ during the high-temperature treatment enhances lateral growth of initial Ge islands and promotes faster formation of continuous Ge nanowires in trenches. The Kolmogorov–Johnson–Mehl–Avrami crystallization model supports our explanation of Ge coalescence. These results provide critical insight into the selective epitaxy of horizontal Ge nanowires on lattice-mismatched Si substrates, which can be translated to other material systems.

## Abstract 2

Schematics on the influence of Ge islands on the formation of continuous nanowires on silicon. In selective area epitaxy, islands with low density and low aspect ratio merge together to produce a continuous Ge nanowire on a silicon substrate.

## Graphical abstract

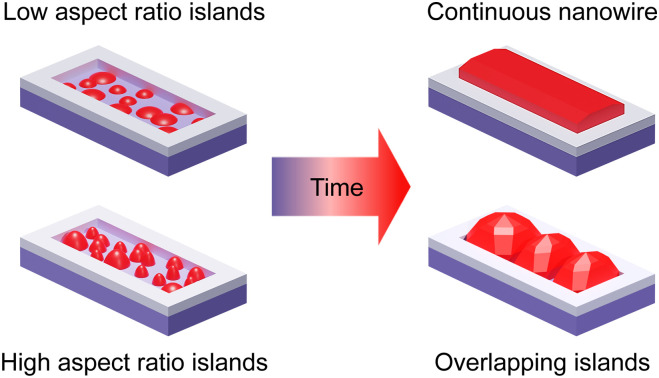

**New concepts**

The selective area epitaxy approach (SAE) has emerged as a means of directly organizing nanowires at the location of future devices in a scalable manner. Realizing this potential requires a complete understanding and control of the initial stages of the epitaxy process. This study reveals, for the first time, the underlying growth mechanism of Ge nanowires during selective area epitaxy and the origin of crystal imperfections. Highlighting the crucial role of pre-growth surface treatment, our results demonstrates that exposure to AsH_3_ during pre-growth surface treatment enhances the lateral growth of initial Ge islands, resulting in a faster formation of continuous nanowires. It also minimizes defect formation. Furthermore, the application of the 2D Kolmogorov–Johnson–Mehl–Avrami (KJMA) crystallization model accurately captures the growth kinetics of island coalescence during SAE, supporting our explanations. These results provide essential insights into the selective epitaxy of horizontal Ge nanowires on lattice-mismatched Si substrates, which can be applied to other material systems.

## 1 Introduction

Site-selective integration of nanowires on a host substrate is essential for the scalability of nanowire devices.^1,2^ In recent years, the selective area epitaxy (SAE) approach has emerged as a means of precisely arranging nanowires in the locations of future devices in a scalable and deterministic manner. This technique uses openings created in a crystalline substrate covered with a mask to obtain nanowires of different geometries. The mask, typically made of oxide material to which growth precursors do not adhere at the growth temperature, restricts nanowire growth to the unmasked regions of the substrate.^3–7^

Compared with conventional free-standing nanowire growth,^8–13^ the SAE approach offers several advantages.^14^ Firstly, the SAE process allows for the direct growth of the nanostructures in locations of future devices. Secondly, the design flexibility of the SAE approach enables the integration of nanoscale devices of varying size and complexity at a wafer scale.^15,16^ Finally, the small interface area between the grown semiconductor and the host substrate limits the formation of interface-related defects, enabling the integration of lattice-mismatched materials.^17,18^ Because of these convenient properties, the SAE approach has been the subject of rapidly increasing interest in the area of electronic and quantum computing applications.^17,19,20^

Among the material platforms available for quantum technologies, holes in Ge nanowires are promising for spin-qubit based quantum computing. This is due to their strong spin–orbit interaction (SOI) and low susceptibility to hyperfine interaction.^21–23^ The strong SOI of holes facilitates fast, electrical manipulation of qubits, while the low susceptibility to hyperfine interactions guarantees long coherence lifetimes. The use of the SAE approach in growing Ge nanowires offers control over the crystalline shape and orientation to cancel out the impact of charge noise and hyperfine interaction on coherence.^24^ Our previous work has demonstrated the SAE of in-plane Ge nanowires and their networks on Si (001) substrates.^4^ Low-temperature electronic transport measurements on nanowire Hall bar devices showed coherent hole transport and a weak anti-localization peak, indicating strong SOI.

This work illuminates the underlying growth mechanism of Ge nanowires in SAE and the origin of crystal imperfections, such as dislocations and stacking faults. It also provides a path to minimizing defect formation. While similar studies have previously explored the SAE of III–V,^25–27^ II–V,^28,29^ and IV–VI^7,30^ compounds, to the best of our knowledge there is no available report on the SAE of Ge nanowires on Si. The SAE of Ge nanowires on Si (001) substrates proceeds through the nucleation and coalescence of Ge islands. We study the effect of the *in situ* surface pretreatment step on the coalescence and crystalline quality of the Ge nanowires. The Kolmogorov–Johnson–Mehl–Avrami's (KJMA) 2D crystallization model is used to capture the growth kinetics of the coalescence process. Finally, we compare the crystal quality and investigate the origin of defects in Ge nanowires using aberration-corrected scanning transmission electron microscopy (STEM). From this point of view, the present study provides a critical insight into the SAE of horizontal Ge nanowires on Si substrates that can be translated to other materials systems.

## 2 Experimental procedures

**Fig. 1:**
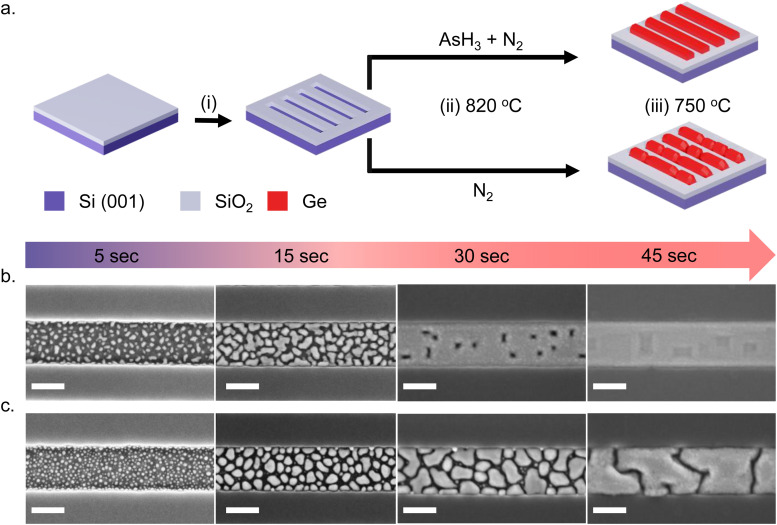
(a) Schematic illustration of the SAE process by MOVPE: (i) substrate patterning step to define trenches in the SiO_2_ mask, (ii) surface pretreatment step at 820 °C to ensure a clean Si (001) surface and (iii) Ge nanowire growth at 750 °C. Nucleation and coalescence of Ge islands during SAE: top view SEM images showing different stages of Ge film growth inside a 250 nm wide trench on Si (001) surface obtained after the surface pretreatment step (b) with and (c) without AsH_3_. The scale bar shown in (b) and (c) indicates 200 nm.

Fig. 1a shows a schematic illustration of the SAE process. We start with an intrinsic Si (001)/SiO_2_ substrate with nanoscale trenches defined along the 〈110〉 crystallographic direction. The substrates investigated in this study consist of an array of 10 parallel nanoscale trenches with a length of 20 μm and a center-to-center spacing of 1 μm. Details of the substrate patterning procedure are provided in our previous publication^4^ and in the ESI.† The substrates are briefly etched in dilute HF solution (1 : 100 dilution with H_2_O) to remove the native surface oxide of silicon inside the trenches and transferred into an Aixtron 200 MOVPE system. The Ge nanowire growth recipe begins with an annealing step at 820 °C – the maximum temperature achievable in our system – to ensure a clean surface inside the nanoscale trenches. From here on, we call this the surface pretreatment step. After the surface pretreatment step, the substrates are cooled down under N_2_ in the absence of AsH_3_ to the nanowire growth temperature of 750 °C in 4 minutes. All growths mentioned in this work are carried out at 30 mbar pressure using isobutyl germane (IBuGe) as the Ge precursor and N_2_ as the carrier gas. The partial pressure of IBuGe is adjusted to deliver a nominal growth rate of 1 nm min^−1^ as calibrated separately on planar GaAs substrates. The growth time varies from 2.5 s to 110 s to obtain the temporal evolution of Ge nanowire growth inside the trenches.

We investigate the effect of the surface pretreatment step on the SAE of Ge by comparing two different procedures. In the first case, the surface pretreatment occurs under AsH_3_ flow (60 sccm) diluted in N_2_ (3500 sccm). In the second case, the substrate pretreatment is performed under N_2_ flow (3500 sccm) without AsH_3_. The process is summarized in Fig. 1a. Using scanning electron microscopy (SEM), atomic force microscopy (AFM), and transmission electron microscopy (TEM), we compare the time evolution and crystalline quality of germanium nanowires obtained on a Si (001) surface with the two different surface pretreatment procedures.

## 3 Results and discussion

Fig. 1 describes the growth behavior of Ge inside the trenches as a function of the growth time and the surface pretreatment step. The top view SEM images in Fig. 1b and c depict the time evolution of Ge growth inside a 250 nm wide trench obtained after the surface pretreatment step with and without AsH_3_, respectively. We provide overview SEM images from the whole array in the ESI† (Fig. S2). Irrespective of the surface pretreatment procedure, the SAE of Ge initiates through the nucleation of multiple small islands inside the trenches, as visible in the SEM images (Fig. 1b and c) after 5 s of growth. However, the surface pretreatment procedure influences the nucleation density and the coalescence process of the Ge islands. Introducing AsH_3_ during the surface pretreatment step (Fig. 1b) lowers the surface density of Ge islands obtained after 5 s of growth. As growth progresses, the Ge islands expand in volume and coalesce, initially forming Ge films with holes. Finally, the holes fill to create continuous Ge films inside the trench over 45 s of growth. A similar growth behavior was observed during the selective growth of Ge on micrometer-sized Si pillars in ref. 31. On the other hand, omitting AsH_3_ during the surface pretreatment step (Fig. 1c) results in a higher density of initial Ge islands after 5 s of growth. As the deposition continues, the Ge islands merge to form larger but more compact islands. However, after 45 s of growth, the overlapping islands no longer merge thereby forming a discontinuous film.

**Fig. 2:**
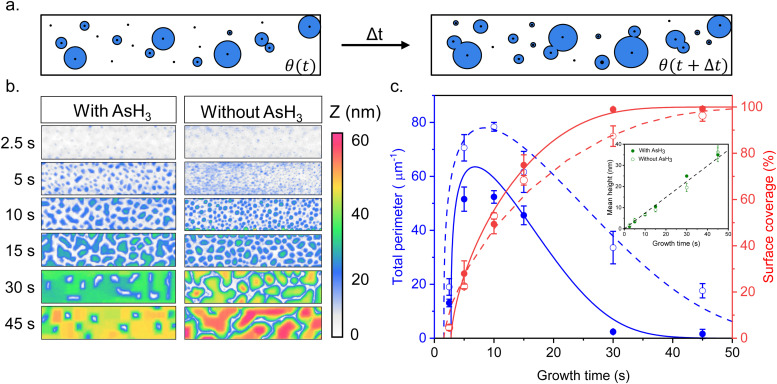
(a) Schematic illustration of the KJMA crystallization model. (b) AFM contour plots comparing the time evolution of a growing Ge film inside the trenches as a function of surface pretreatment conditions. (c) Perimeter per surface area (blue color) and surface coverage (red color) of Ge film grown by MOVPE inside the patterned oxide trenches on Si (001) surface with (filled circle) and without (empty circle) AsH_3_ during the surface pretreatment step, fitted by the model (solid lines) with the parameters summarized in Table 1. The inset figure shows the time evolution of the mean height of the Ge film inside the trenches.

In order to shed more light on the difference in the time evolution of Ge growth, we model the coalescence process of Ge islands. Growth modeling of thin films starting from three-dimensional (3D) islands, that rapidly coalesce into continuous film inside the trenches in SAE, is not a simple problem because the morphology of the film in the coalescence stage cannot be described in terms of the size, shape, and surface density of individual islands. Solid-like coalescence of crystal islands is usually treated using the KJMA crystallization model.^32–34^ In the case of two-dimensional (2D) crystallization,^35,36^ it provides the time-dependent surface coverage *θ*(*t*) and perimeter of the crystallization front per unit area of the surface *P*(*t*) explicitly. A schematic illustration of the 2D KJMA model is shown in Fig. 2a.

The general expressions of the KJMA model are given by1
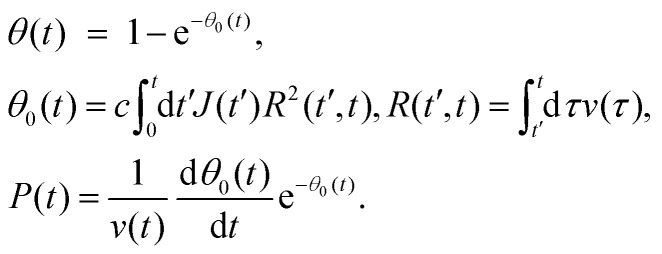


Here, *J*(*t*) is the nucleation rate, *v*(*t*) is the lateral growth rate of Ge islands, *R*(*t*′,*t*) is the linear size of the island base at time *t* for an island nucleating (with zero size) at time *t*′, *c* is a 2D shape constant and *θ*_0_(*t*) is the extended filling factor which would be observed on a surface if all the merged Ge islands were again separated.^32–35,37^*v*(*t*) is the lateral growth rate of Ge islands.

We use this model to understand the growth kinetics of Ge films shown in Fig. 1 in terms of the time-dependent mean height *H*(*t*), perimeter *P*(*t*), and coverage *θ*(*t*) of the Ge film. For this purpose, we perform AFM measurements on the time evolution samples from Fig. 1 and compare the effect of surface pretreatment conditions. The results are presented in Fig. 2. Fig. 2b corresponds to representative AFM images of Ge films inside a 250 nm wide trench as a function of growth time for the two surface pretreatment conditions. The AFM measurements are acquired from a scan window of 1 μm × 250 nm with the zero height set to the Si (001) substrate. Using the 2-D projections of the AFM data presented in Fig. 2b, we quantify the time-dependent mean height *H*(*t*), perimeter per unit surface area *P*(*t*), and the total surface coverage *θ*(*t*) of the Ge film. Fig. 2c summarizes the time-dependence of the surface coverage (red color) and perimeter per unit surface area (blue color) for the two pretreatment conditions. The inset figure shows the time evolution of the mean height of the Ge film inside the trenches. More details of the statistical analysis are in the ESI.†

One important observation is that the measured surface density of separated Ge islands reaches a very high value after 5 s of growth. We measure an island surface density of 5 × 10^10^ cm^−2^ for samples where the surface pretreatment is performed with AsH_3_ and 2 × 10^12^ cm^−2^ in the absence of AsH_3_. In addition, the perimeter of Ge film reaches its maximum around 5 s to 10 s in both cases, corresponding to the beginning of the coalescence (Fig. 2c). Therefore, we use the approximation of instantaneous nucleation at the moment *t*_0_ where 3D growth starts:^35,37^*J*(*t*) = *Nδ*(*t* − *t*_0_), with *N* as the island surface density. In this case, the surface coverage and perimeter given by eqn (1) can be presented as functions of *R* in the form:2
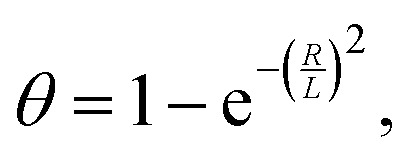
3
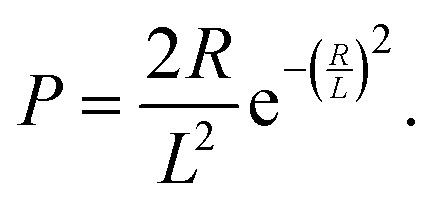
The separation between Ge islands (*L*) is related to their surface density in the pre-coalescence stage *N* according to *L*^2^ = 1/(*cN*).

To describe the time dependence of the coverage and perimeter (Fig. 2c), we need to relate the base size of Ge islands *R* to time *t*. This requires knowledge of the growth kinetics of individual Ge islands in the general case.^37,38^ In our MOVPE growth, this process can be simplified because the mean height of the Ge film (*H*) is linear in time for all *t* (Fig. 2c inset). The mean height (*H*) of the Ge layer equals *θh*, with *h* = *αR* as the mean height of Ge islands (including the merged islands) and *α* = *h*/*R* is the aspect ratio. Using the linear relationship

4 *θαR* = *v*_2D_(*t* − *t*_0_),

and eqn (2) for *θ*(*R*), the required *R*(*t*) dependence is obtained in the form5
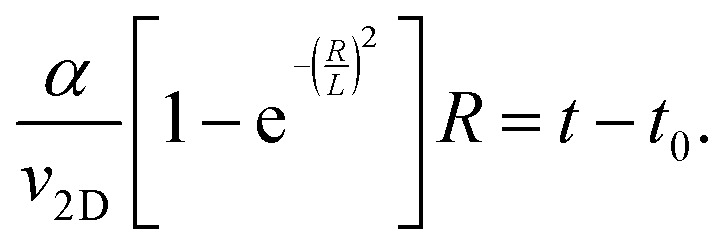
According to the data, the 2D growth rate of Ge *v*_2D_ is unaffected by the surface pretreatment condition and equals 0.745 nm s^−1^ in both cases. With known *v*_2D_, eqn (2), (3), and (5) implicitly provide the time dependence of the coverage and perimeter, with the fitting parameters *α* and *L*.

We now explain the growth kinetics of the Ge islands presented in Fig. 2c using the KJMA model. The lines in Fig. 2c show the fits to the data obtained from eqn (2), (3), and (5) with the parameters given in Table 1, which describe the observed morphological evolution of the Ge films. According to the data, the coverage of the Ge film increases, and the perimeter decreases significantly faster when we use AsH_3_ during the surface pretreatment step. On the other hand, the measured density of separated Ge islands in the pre-coalescence stage is higher without AsH_3_, corresponding to a larger maximum perimeter of the islands. Thus, we can conclude that the introduction of AsH_3_ in the surface pretreatment step reduces Ge nucleation and enhances Ge lateral growth with respect to its vertical growth, leading to a faster formation of a continuous Ge film inside the trenches.

**Table 1:** Parameters of Ge growth on Si (001) surface as a function of surface pretreatment condition

Surface pretreatment step	2D growth rate *v*_2D_ (nm s^−1^)	Nucleation time *t*_0_ (s)	Aspect ratio (*α*)	Separation between islands *L* (nm)	Maximum surface density *N* (μm^−2^)
With AsH_3_	0.745	2.8	0.8	13.5	1370
Without AsH_3_	0.745	1.6	1.65	10.5	2268

The KJMA model also gives insights into the aspect ratio (*α*) and nucleation time (*t*_0_) for the Ge islands as a function of the surface pretreatment step (Table 1). For the surface pretreatment condition without AsH_3_, the nucleation of the 3D Ge islands occurs after *t*_0_ = 1.6 s, and the nuclei have an aspect ratio of 1.65. The presence of AsH_3_ during the surface pretreatment step delays the nucleation of 3D islands to *t*_0_ = 2.8 s and results in islands with a smaller aspect ratio, *α* = 0.8. The fitting values of the separation *L* yield higher maximum surface densities of Ge islands (calculated for the square base of Ge islands at *c* = 2) compared to the measured values after 5 s growth as shown in Table 1. Two reasons may cause this. First, the measured island densities at *t* = 5 s may not correspond to their maximum values. Second, the assumed instantaneous nucleation is an approximation, even if the nucleation stage is short compared to the total time of film growth.^37^

We attribute the differences in the nucleation density and the growth kinetics of Ge islands to the chemical nature of the Si (001) surface. According to ref. 39 and 40, introducing AsH_3_ during the surface pretreatment step serves two purposes. Firstly, at the annealing temperature of 820 °C used in this study, it ensures deoxidation of the silicon substrate. The H^+^ species produced by the dissociation of AsH_3_,^41^ remove carbon and oxygen from the Si surface.^42,43^ This is believed to happen through a surface reaction that results in volatile species such as CH_4_ and H_2_O.^44^ Secondly, As atoms passivate Si dangling bonds, providing a stable As-terminated Si surface for the growth of Ge.^45,46^ Under these conditions, Ge growth on the Si (001) surface follows the typical Stanski–Krastanov (SK) growth mode, where Ge islands are formed by the spontaneous transformation of an initially formed 2D wetting layer of Ge.^47^ The measured island surface density of 5 × 10^10^ cm^−2^ is within the range of values commonly reported for the SK growth of Ge on the Si (001) surface.^48,49^ In contrast, when AsH_3_ is omitted, we expect the presence of patches of residual oxide on the Si surface. This can be due to re-oxidation of the surface after the HF etch.^50^ The presence of an oxide drastically alters the growth mode. The growth of Ge on an oxidized Si surface follows the Volmer–Weber (VW) growth mode with the direct nucleation of a 3D island at the voids present in the SiO_2_ layer, without forming a wetting layer.^51–53^ It is interesting to note that Ge islands obtained on a Si surface covered with a thin oxide layer (0.5 nm to 1.2 nm thick) typically feature a very high nucleation density (2 × 10^12^ cm^−2^)^51–53^ and an aspect ratio close to 1.6 similar to the values observed in this study,^53^ which supports our claim. Based on this, we conclude that the calculated delay in the nucleation of the 3D island after the surface pre-treatment with AsH_3_ can be related to the formation of the wetting layer during the SK growth.

The presence of a residual oxide between the Ge islands also explains the differences we observe in the growth kinetics of the Ge islands. The low surface energy of the SiO_2_ (*γ*_SiO_2__ ≈ 0.4 J m^−2^)^54^ compared with the surface energy of Ge (*γ*_Ge_ ≈ 1.05 to 1.71 J m^−2^)^55^ and the Ge–SiO_2_ interfacial energy (*γ*_Ge–SiO_2__ ≈ 1 J m^−2^),^56^ promotes the vertical growth of Ge islands on SiO_2_ surface to minimize the total surface free energy. On the other hand, the Ge islands obtained on an AsH_3_ treated Si surface follow the SK growth mode and form on a 2D wetting layer of Ge.^47^ Here, the islands can expand laterally over the wetting layer until they reach an equilibrium state dictated by the strain energy resulting from lattice mismatch.^57–59^ Therefore, the higher aspect ratio and the enhanced vertical growth rate of Ge island observed after the surface pretreatment step without AsH_3_ are coherent with the presence of a residual oxide layer on the Si surface.

**Fig. 3:**
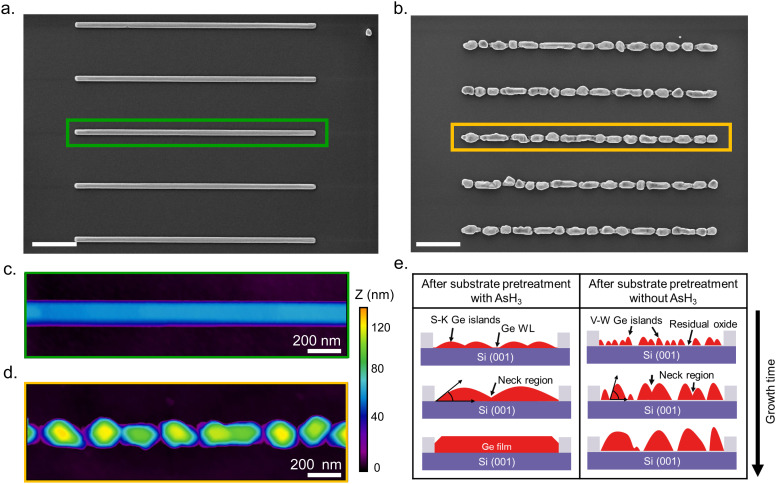
Effect of surface pretreatment condition on the continuity of Ge nanowires. (a) Top view SEM image of continuous Ge nanowires obtained after the surface pretreatment step with AsH_3_. (b) Top view SEM image of discontinuous Ge islands obtained after the surface pretreatment step without AsH_3_. The SEM images are acquired from an array of five SiO_2_ trenches with a nominal width of 80 nm after 110 s of growth. (c) AFM counterplot acquired from the continuous nanowire located at the center of the array shown in (a). (d) AFM counterplot acquired from the discontinuous Ge islands located at the center of the array shown in (b). (e) Schematic illustration of the coalescence process of the Ge islands as a function of the surface pretreatment condition. The scale bar shown in (a) and (b) indicates 1 μm.

Having explained the differences in the growth kinetics of the Ge islands as a function of the surface pretreatment step, we now compare the morphology, chemical composition, and crystal quality of the Ge nanowires. Fig. 3 contains representative SEM and AFM images of the Ge nanostructures obtained as a function of the surface pretreatment step. The SEM images are acquired from an array of five parallel SiO_2_ trenches with a nominal width of 80 nm after 110 s of Ge growth. Fig. 3a shows the top view SEM image of an array of five continuous Ge nanowires obtained after the surface pretreatment step with AsH_3_. A representative AFM counterplot of a continuous nanowire is provided in Fig. 3c. Fig. 3b shows the top view SEM images of the discontinuous Ge islands inside the SiO_2_ trenches obtained after the surface pretreatment step without AsH_3_. A representative AFM counterplot of the discontinuous Ge islands is provided in Fig. 3d. The observed trend is consistent for Ge nanowires obtained from SiO_2_ trenches of varying nominal width (Fig. S4, ESI†). Both the SEM and AFM images indicate that the surface pretreatment step directly influences the continuity of SAE Ge nanowires. Ge nanowires with dimensions defined by the substrate patterning step are obtained only after the surface pretreatment step with AsH_3_ (Fig. 3a and c). By contrast, the omission of AsH_3_ (Fig. 3b and d) during the surface pretreatment step results in discontinuous Ge islands inside the trenches. The observed differences in the coalescence process are explained using Nicholas and Mullikan's model.^60,61^ A schematic illustration of our understanding of the coalescence process is provided in Fig. 3e. The coalescence of two crystalline islands into one larger island occurs by the surface diffusion of atoms caused by differences in the radius of curvature.^61^ The neck region formed at the point of impingement between two nearby islands gets preferentially filled by atoms diffusing down from the top surface of the islands.^62^ According to this description, the coalescence time between two impinging islands is quantitatively given by eqn (6), where *R* is the radius of the smaller island in the coalescing pair, and *B* is the coalescence strength.^62,63^6
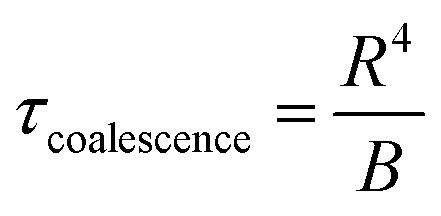


Once the neck region has been filled by the coalescence of islands, surface energy minimization brings the newly formed island to an equilibrium shape.^62,63^ The presence of an oxide layer on the Si surface after the surface pretreatment step without AsH_3_ influences the equilibrium shape and favors the formation of islands with high aspect ratios (*α*). As a result, the islands evolve into a more compact shape, as observed in Fig. 1b. Finally, the size of the islands (*R*) becomes prohibitively large for island coalescence to occur, and all the subsequent deposition of the atoms will feed the island growth, forming interconnected islands without coalescence as observed in Fig. 3b and d.

**Fig. 4:**
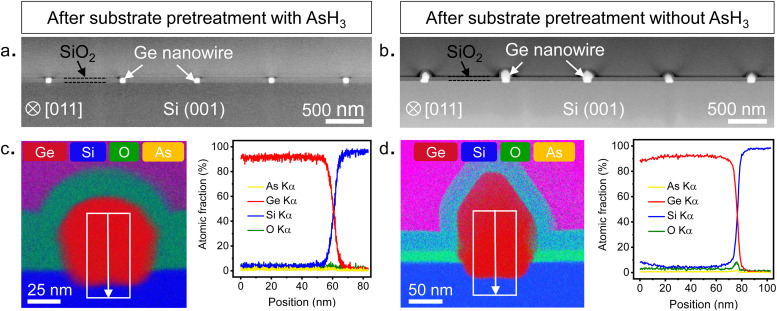
Compositional assessment of the Ge nanowires. HAADF-STEM micrograph of a cross-section cut made perpendicular to the in-plane orientation of five parallel nanowires obtained after surface pretreatment with AsH_3_ (a) and without AsH_3_ (b), respectively. (c) and (d) show the chemical composition of the Ge nanowires. (c) STEM-EDX elemental mapping showing the elemental distribution in Ge nanowires obtained after surface pretreatment with AsH_3_. The accompanying EDX line scan profile reveals the sharpness interface between the Ge nanowire and the Si substrate. (d) STEM-EDX elemental mapping showing the elemental distribution in Ge nanowires obtained after surface pretreatment without AsH_3_. The EDX line scan profile shows the presence of an oxide layer at the Ge nanowire/Si substrate interface.

To evaluate the chemical composition of the nanowires, we prepared cross-sectional cuts perpendicular to the growth direction of the nanowires shown in Fig. 2 and examined them using a STEM. Further details on sample preparation for STEM are provided in the ESI.† The low magnification HAADF STEM images shown in Fig. 4a and b provide an overview of the cross-sections of five different nanowires obtained after surface pretreatment with and without AsH_3_, respectively. The cross-sections of the nanowires were analyzed by scanning transmission electron microscopy with energy dispersive X-ray spectroscopy (STEM-EDX) to quantitatively evaluate the elemental composition and distribution in the nanowires. The results of the STEM-EDX elemental mapping are shown in Fig. 4c and d. STEM-EDX elemental mapping profiles of individual elements are provided in the ESI† (Fig. S7 and S8). For the surface pretreatment condition with AsH_3_ (Fig. 4c), the chemical analysis by STEM-EDX confirms the Ge-rich composition of the nanowire and reveals the sharpness of the interface with the Si substrate. The EDX line scan profile taken along the nanowire-substrate interface shows a diffused interface with a slight migration of Si into the Ge up to a distance of 5 nm from the interface. The observed diffuse interface could also be due to the roughness of the Si surface created during the substrate fabrication (Fig. S3, ESI†). Qualitatively, we were also able to observe traces of As atoms within the Ge nanowires in the individual EDX composition map of As (Fig. S7, ESI†). However, we were not able to quantify them in the EDX line scans (Fig. 4c) or by collecting spectra from different points (Fig. S7, ESI†). From this, we can conclude that the arsenic doping in the Ge nanowires is minimal and within the detection limit of the STEM-EDX technique used in this study. In contrast, the Ge nanowires obtained after the surface pretreatment without AsH_3_ showed the presence of oxygen at the Ge/Si interface (Fig. 4c). The oxide layer was observed in all nanowire cross-sections analyzed in this study (Fig. S8, ESI†). Further details on the quality of the interfaces are described in the following sections.

**Fig. 5:**
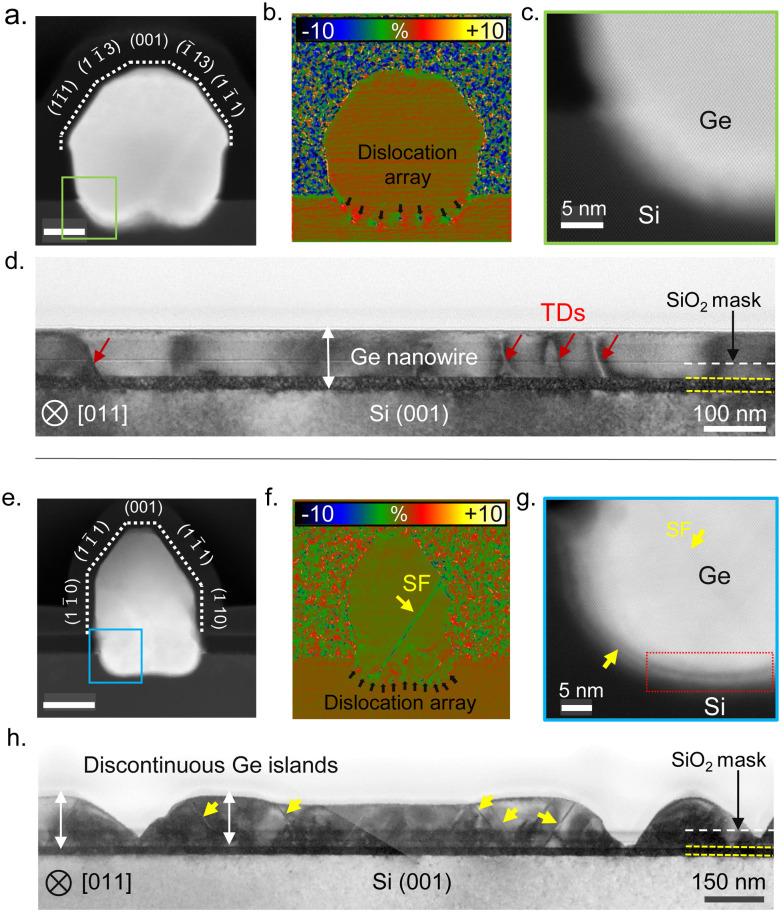
(a)–(d) Present cross-sectional morphology and crystal quality of the Ge nanowires obtained after the surface pretreatment condition with AsH_3_. (a) Atomic-resolution HAADF-STEM micrograph of a nanowire cross-section cut made perpendicular to the in-plane orientation. (b) GPA strain analysis map of the cross-section presented in panel (a), displaying mean plane rotation in the {111} plane. (c) Atomic-resolution HAADF-STEM image of the region marked in green in panel (a), revealing the presence of a diffused interface between the Ge nanowire and the Si substrate. (d) Bright-field TEM image of a cross-section cut made parallel to the in-plane orientation of the nanowire. The yellow dotted lines represent the moiré pattern, marking the interface between the Ge nanowire and the Si substrate. (e)–(h) Present the cross-sectional morphology and crystal quality of the Ge nanowires obtained after the surface pretreatment condition without AsH_3_. (e) Atomic-resolution HAADF-STEM micrograph of a nanowire cross-section cut made perpendicular to the in-plane orientation. (f) GPA strain analysis map of the cross-section presented in panel (e), indicating mean plane rotation in the {111} plane. (g) Atomic resolution HAADF STEM image of the region marked in blue in panel (e), revealing the presence of an interfacial amorphous layer (red box) at the interface between the Ge nanowire and the Si substrate. (h) Bright-field TEM image of a cross-section cut made parallel to the in-plane orientation of the nanowire.

To assess the crystal quality, we analyzed the nanowire cross-sections presented in Fig. 4 using aberration-corrected STEM. Fig. 5 presents a comparison between the Ge nanowires obtained after the surface pretreatment step with and without AsH_3_ in terms of the cross-sectional morphology (a) and (e), strain distribution (b) and (f), and nanowire–substrate interface (c) and (g). When AsH_3_ is included in the surface pretreatment, nanowires exhibit uniform height and cross-sectional morphology, as depicted in Fig. 4a. We show an atomic resolution HAADF STEM image of a representative nanowire cross-section in Fig. 5a. The dotted lines in Fig. 5a indicate the cross-sectional morphology of the nanowire and are defined by facets belonging to the {110}, {111}, {001}, and {113} families, as in our previous work.^4^ We also observe a small amount of lateral growth along the [1−10] and [−110] directions above the oxide mask. Geometric phase analysis (GPA) of the nanowire cross-section (Fig. 5b) reveals plastic relaxation of the lattice mismatch by creating a periodic array of the misfit dislocation at the Ge/Si interface. In the present case, it seems that all strain is relaxed through plastic relaxation, with no presence of induced plane rotation at the lateral basis of the nanowire (elastic relaxation), as commonly observed in other nanowire systems.^20,64,65^ The magnified image of the Ge/Si interface in Fig. 5c shows a diffused interface indicative of the alloying between Si and Ge. In contrast, skipping AsH_3_ during the surface pretreatment step results in Ge islands with non-uniform height and cross-sectional morphology as observed in the HAADF STEM images (Fig. 4b and 5e). GPA analysis of the nanowire cross-section (Fig. 5f) shows the presence of stacking faults in addition to misfit dislocations. Finally, the magnified image of the Ge/Si interface presented in Fig. 5g shows patches of amorphous interfacial layer (white arrows). The Ge nanowire maintains an epitaxial relationship with the substrate but contains twin boundaries and stacking faults.

We further investigate the crystal quality along the length of the nanowire by preparing cross-sectional cuts parallel to the in-plane orientation of the nanowire. Fig. 5d and h show the bright field TEM image of cross-section cuts made parallel to the in-plane orientation of Ge nanowire obtained after surface pretreatment with and without AsH_3_, respectively. Although the nanowire growth occurs by the nucleation and coalescence of Ge islands, Ge nanowires obtained after the surface pretreatment step with AsH_3_ exhibited excellent crystal quality with few threading dislocations (red arrows in Fig. 5d). The contrast variation observed along the cross section in Fig. 5d originates from a strain developed during the coalescence of islands.^66,67^ Notably, the crystal quality that we observe in the present work is improved with respect to our previous work, where nanowire growth was carried out at 700 °C and the surface pretreatment step was performed at a lower temperature of 780 °C.^4^ We attribute the improvement in the crystal quality to the optimization of the surface pretreatment step and alloying of the interface due to a higher growth temperature. In contrast, skipping AsH_3_ during the surface pretreatment results in discontinuous Ge islands with a high density of twins and stacking faults as seen in Fig. 5e and h. Most defects start at the interface between the Ge islands and Si and extend into the upper part. The absence of extended planar defects in Ge nanowire samples grown after the surface pretreatment with AsH_3_ (Fig. 5a and d) suggests the residual oxide layer causes their nucleation. Ge islands nucleating on voids in the SiO_2_ layers can have a translation or tilt mismatch between adjacent islands. The coalescence of islands with translation mismatch generates stacking faults and twins in the Ge layer. In addition, the stress resulting from the thermal expansion mismatch between Ge and SiO_2_, or during the impingement of islands with a high aspect ratio, can also induce deformation twins in these structures.^66^

## 4. Conclusion

In summary, we investigate the selective area epitaxy of horizontal Ge nanowires on Si (001) substrates using MOVPE and demonstrate the impact of the use of AsH_3_ in the surface pretreatment step on the nanowire growth and crystal quality. The SAE of Ge nanowires proceeds by the nucleation and coalescence of Ge islands, and the introduction of AsH_3_ during the surface pretreatment step modifies the nucleation kinetics and coalescence process of the Ge islands. Using the KJMA crystallization model for the 2D projection of Ge islands, we model the coalescence process of Ge islands to understand the differences in nucleation and growth kinetics. Our results indicate that the epitaxy of Ge islands on the Si surface after the surface pretreatment step using AsH_3_ follows the Stanski–Krastanov growth mode. In contrast, skipping the AsH_3_ during the surface pre-treatment step results in the Volmer–Weber growth of Ge islands. We also show that using AsH_3_ during the surface pretreatment step enhances the lateral growth of the Ge islands, leading to a faster formation of continuous Ge film inside the trenches. Skipping AsH_3_ during the surface pretreatment step results in discontinuous Ge islands for the same growth time. We attribute the observed difference in the nucleation and growth to the differences in the chemical nature of the Si surface. The presence of AsH_3_ during the surface pretreatment step ensures the complete removal of native oxide of silicon and provides a clean As-terminated Si (001) surface for Ge epitaxy. Finally, Ge islands grown on the Si surface after the surface pretreatment step without AsH_3_ show the presence of a high density of planar defects (stacking faults and twins). On the contrary, planar defects are minimal on Ge nanowire grown with the AsH_3_ surface pretreatment. In this respect, this work demonstrates the importance of a thorough understanding of the chemical nature of the Si (001) surface and their control through *in situ* surface pretreatment to optimize the crystal quality of the SAE Ge nanowires.

## Author contributions

S. P. R. was responsible for substrate fabrication, nanowire growth and morphological characterization, BF-TEM analysis, STEM EDX compositional maps, and the fabrication of hall bar devices for electrical characterisation. J. R. conducted all the AFM measurements and performed data analysis. V. G. D. handled the KJMA modeling of the coalescence process and wrote the corresponding sections of the manuscript. S. M. and J. A. conducted aberration-corrected scanning transmission electron microscopy studies. A. R. contributed to optimizing the Ge nanowire growth recipes and maintaining the MOCVD facility. A. M. was responsible for fabricating the top gated device and conducting magneto transport measurements. A. F. i. M. was responsible for funding acquisition, resources, and project supervision. S. P. R. conceptualized the work and wrote the manuscript together with A. F. i. M., with inputs from V. G. D. and J. A. All authors commented on the work and approved the final version of the manuscript.

## Conflicts of interest

There are no conflicts to declare.

## Supplementary Material

NH-009-D3NH00573A-s001
