# Supplementary material for: Control of Ge island coalescence for the formation of nanowires on silicon
Source: Nanoscale Horiz. 2024 Feb 12;9(4):555–65. doi: 10.1039/d3nh00573a (PMC10962639; doi:10.1039/d3nh00573a)
Supplement: NH-009-D3NH00573A-s001 [file NH-009-D3NH00573A-s001.pdf]

Electronic Supplementary Material (ESI) for Nanoscale Horizons.
This journal is © The Royal Society of Chemistry 2024

Supporting information:

Control of Ge island coalescence for the formation of nanowires on silicon.

Santhanu Panikar Ramanandan, [†] Joel Reñé, [†] Alban Morelle,[‡] Sara Martí-Sánche,[¶] Alok Rudra, [†,§] Jordi Arbiol, [¶,∥] Vladimir Dubrovskii, [⊥] and Anna Fontcuberta i Morral [*,†,§,#]

† *Laboratory of Semiconductor Materials, Institute of Materials, Ecole Polytechnique Fédérale de Lausanne (EPFL), Lausanne 1015, Switzerland*

‡ *Solid State Laboratory, ETH Zurich, 8093 Zurich, Switzerland*

¶ *Catalan Institute of Nanoscience and Nanotechnology (ICN2), CSIC and BIST, Campus UAB, Bellaterra, Barcelona, Catalonia, Spain*

§ *Institute of Physics, Faculty of Basic Sciences, Ecole Polytechnique Fédérale de Lausanne (EPFL), Lausanne 1015, Switzerland*

∥ *ICREA, Pg. Lluís Companys 23, 08010 Barcelona, Catalonia, Spain*

⊥ *Faculty of Physics, St. Petersburg State University, Universitetskaya Embankment 13B,199034 St. Petersburg, Russia*

Center for Quantum Science and Engineering, Ecole Polytechnique Fédérale de Lausanne (EPFL), Lausanne 1015, Switzerland

E-mail: anna.fontcuberta-morral@epfl.ch

1. Substrate preparation

Germanium nanowires were grown on a quarter of a 2-inch intrinsic Si (001) substrate with a resistivity of 5000 Ohm-cm. Samples were cleaned using the standard RCA procedure before the deposition of the dielectric mask layer. We used 25 nm thick thermally grown Si dry oxide as the dielectric material. Slits of nominal width varying from 50 nm to 250 nm and length varying from 2 µm to 20 µm were defined on the dielectric mask by e-beam lithography with a 175 nm thick e-beam resist (50 % ZEP). Reactive ion etching (RIE) with a mixture of CHF_3/SF_6 gases was used to transfer the patterns to the dielectric mask. After the RIE step, the substrates were sonicated in acetone (4 mins) and IPA (4 mins) to strip the e-beam resist. To remove any left-over organic residues from resist stripping, the samples were exposed to 600 W oxygen plasma for 10 mins. Finally, a short dip in diluted HF solution (1:10) for 10 secs was performed to remove the left-out SiO_2 layer and native oxide of silicon before the nanowire growth. A schematic illustration of the substrate patterning process is shown in Figure S1.

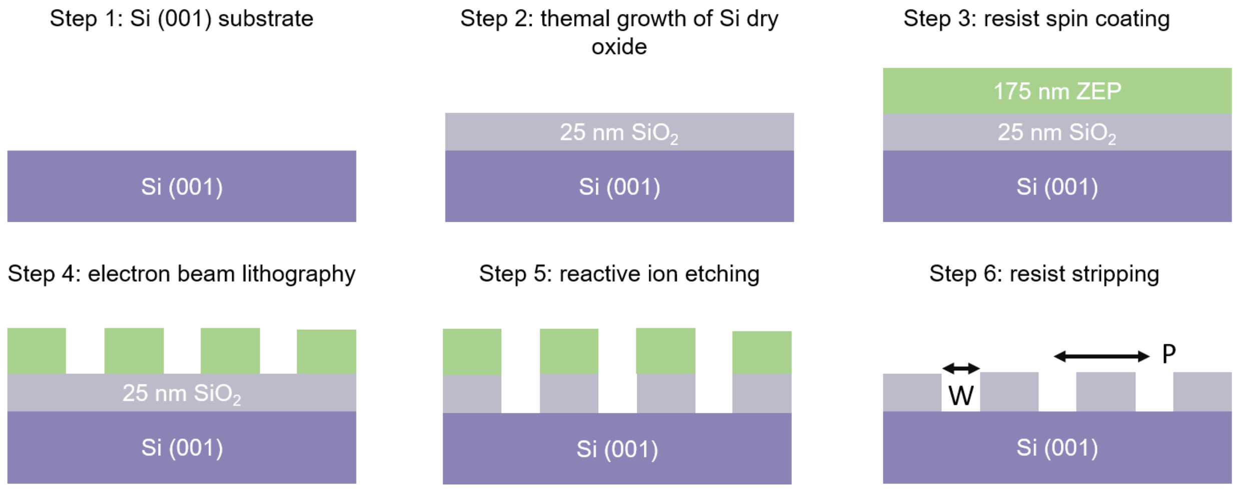

Figure S1: Schematic illustration of the substrate patterning process.

2. Selective area epitaxy (SAE) of germanium

Selective area epitaxy (SAE) of germanium nanowires was carried out using an AIX 200 horizontal metalorganic vapor phase epitaxy (MOVPE) system from Aixtron. Before the nanowire growth, the substrates were thermally annealed at 820 ^oC for 20 mins. The thermal annealing step in the manuscript is called the surface pretreatment step, and we have investigated the effect of two surface pretreatment conditions. After the surface pretreatment step, the substrates were cooled to the growth temperature in N_2 flow in absence of AsH_3. SAE of Ge nanowires was conducted at a reactor temperature of 750 ^oC using isobutyl germane as the precursor molecule and N_2 as the carrier gas. The precursor flow rate was kept at 1 sccm diluted in 3500 sccm of N_2, and the reactor pressure was kept at 30 mbar. The nanowire growths were performed typically for 2.5 s to 2 min, depending on the width of the slits. Top view SEM image of the time evolution of the Ge nanowires as a function of the surface pretreatment step is shown in Figure S2 mentioned above.

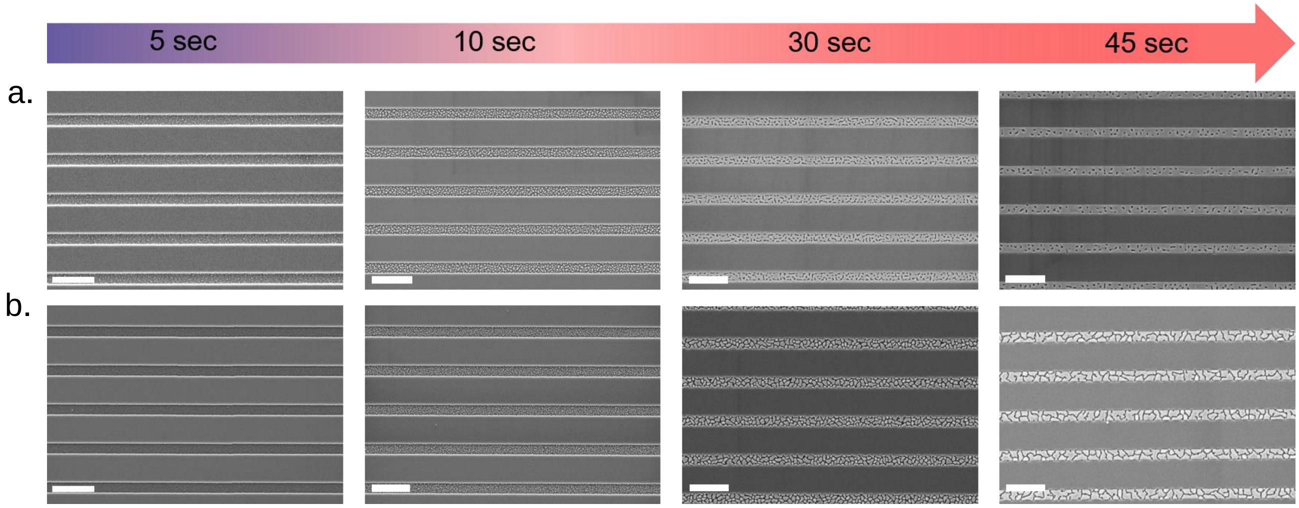

Figure S2: Overview SEM images from the whole array of nanowires depicting the effect of surface pretreatment on the time evolution of Ge nanowires. Time evolution of the Ge nanowires obtained after the surface pretreatment step (a) with AsH_3 and (b) without AsH_3.The scale bar indicates 200 nm.

3. Atomic force microscopy (AFM) study and data processing

Atomic force microscopy was performed using a Bruker FastScan AFM with a Bruker ScanAsyst-Fluid + tip. The AFM scans were performed at a scan rate of 1 Hz using the ScanAsyst mode. The scan resolution was kept at 1024×1024 pixels. The raw AFM data is processed by Gwyddion software. The time-dependent mean height H(t), perimeter P(t), and coverage θ(t) presented in Figure 2 in the main text were extracted from the AFM profiles by setting the mean zero height to the Si substrate.

Si substrate level calibration: The Si substrate level calibration in this work was done in two ways. For the samples where the surface coverage of the Ge film was less than 80 % (for growth times less than 30 seconds), the substrate level was identified by fitting a bimodal Gaussian distribution to the histogram of the AFM height profile acquired from the trench. The centre of the lower Gaussian was then used as the substrate level (zero height in the AFM scans). For samples with a surface coverage of Ge higher than 80 %, the substrate level calibration was done by performing a separate scan on a different growth substrate from the same wafer. The substrate level was identified by fitting the Gaussian distribution to a histogram of the height profile.

Detailing of the islands: After identifying the substrate levels, we utilized the mark grains by threshold option in the Gwyddion software to detail the Ge islands and calculate the perimeter P(t) and coverage θ(t). We used the FWHM of the lower Gaussian as the threshold value.

AFM scans of the growth substrates after the substrate pretreatment step are presented in Figure S3. All the growth substrates used in this study had a surface roughness value of around 1.7 nm. The observed roughness is created during the reactive ion etching step during the growth substrate fabrication. We obtained similar surface roughness values for both of this work's two substrate pretreatment conditions.

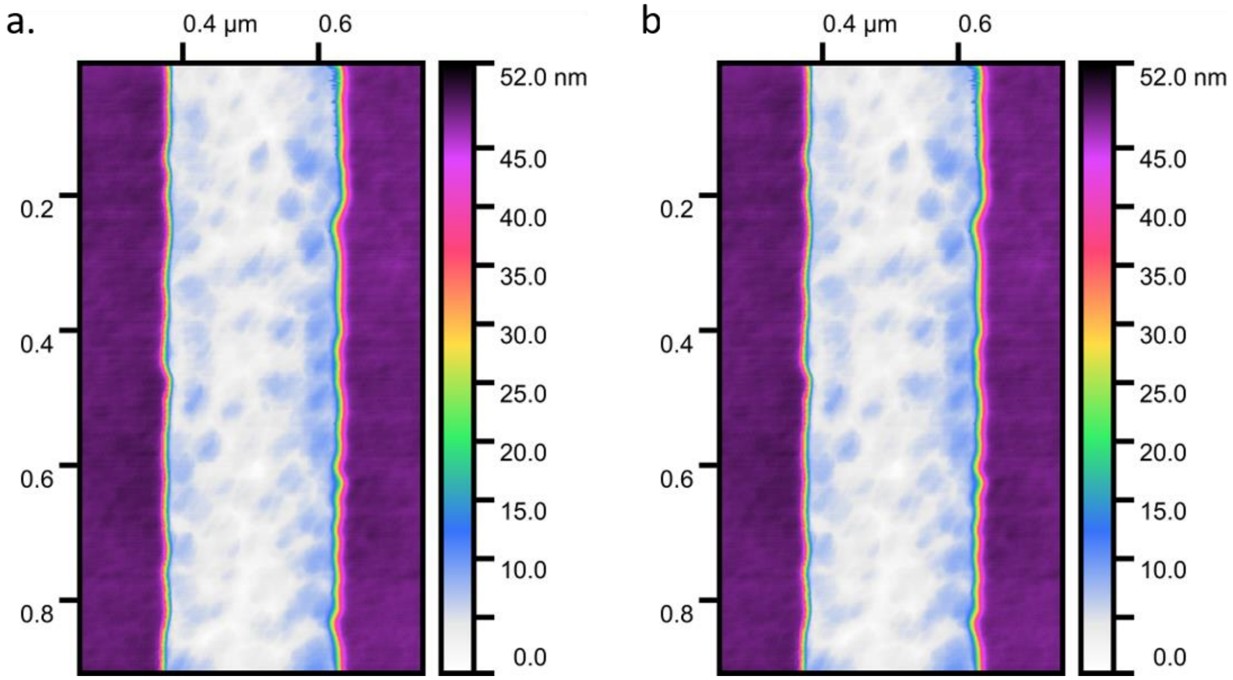

Figure S3: AFM images of the empty growth substrate after the surface pretreatment step (a) with AsH_3 and (b) without AsH_3. The AFM scan is acquired from a SiO_2 trench of nominal width 250 nm and length 20 µm.

4. Surface pretreatment on the continuity of the Ge nanostructures

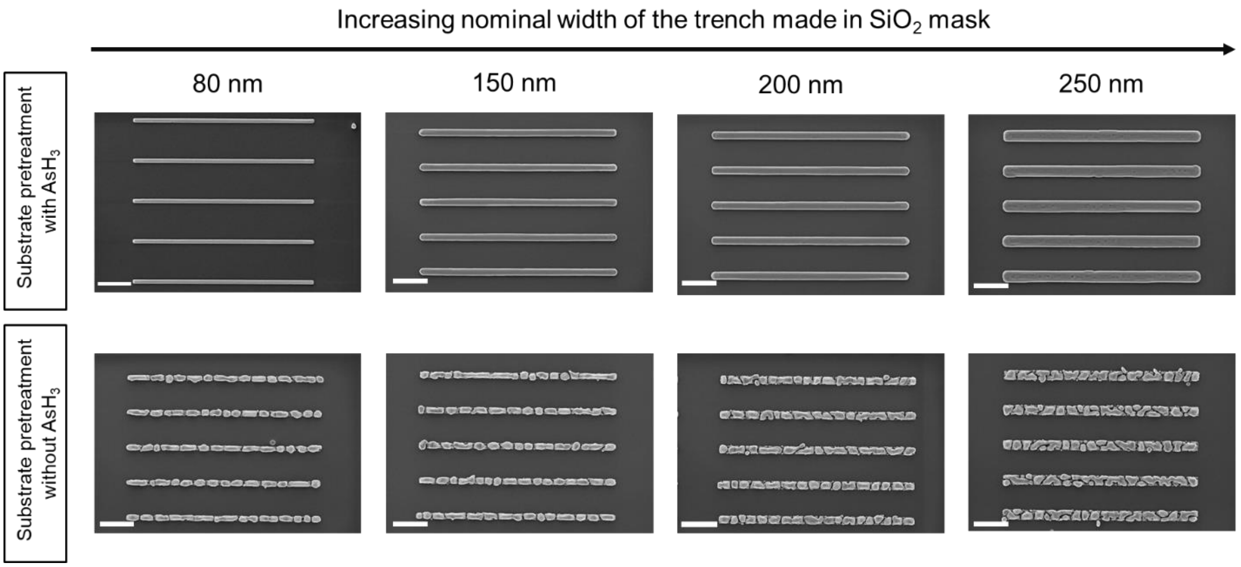

Figure S4: Top-view SEM images of Ge nanostructures inside SiO_2 trenches of varying widths obtained after 110 seconds of growth. The top panel contains SEM images of continuous Ge nanowires obtained after the surface pretreatment step with AsH_3. The bottom panel shows SEM images of discontinuous Ge islands obtained after the surface pretreatment step without AsH_3. The scale bar indicates 1 µm. As mentioned in the manuscript, continuous Ge nanowires are only obtained after the surface pretreatment step with AsH_3.

5. Transmission electron microscopy (TEM) studies

TEM lamellas (longitudinal and perpendicular cross-sections) were prepared using Zeiss NVision 40 focused ion beam (FIB). The germanium nanowires were covered by a thin layer (30 nm thickness) of SiO_2/Si_3N_4 to prevent any surface damage during TEM lamella preparation. The structural quality of the prepared lamellas was investigated using FEI Talos transmission electron microscopy operating at 200kV. Figures S5 and S6 present the representative bright-field (BF) and high-resolution (HR) TEM images of Ge nanowires grown with and without AsH_3 during the surface pretreatment step. As mentioned in the manuscript, Ge nanowires obtained after the surface pretreatment step with AsH_3 show a uniform cross-sectional morphology compared to the nanowires obtained after the surface pretreatment step without AsH_3. The Ge nanowire samples obtained after the surface pretreatment step without AsH_3 also showed a high density of planar defects.

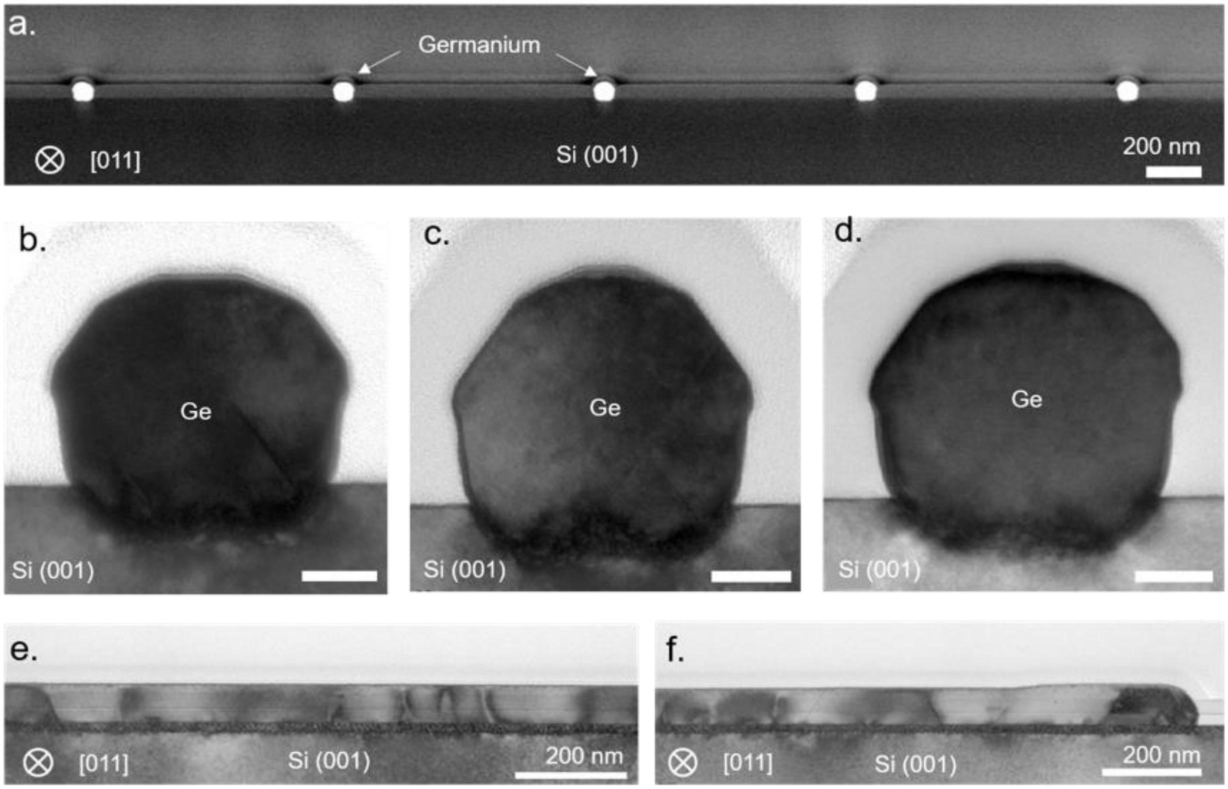

Figure S5: Transmission electron microscopy image of the Ge nanowires obtained after the surface pretreatment step with AsH_3. (a) HAADF STEM image of the Ge nanowire cross-section providing an overview of the cross-sectional morphology. (b-d) BF TEM image of three nanowire cross sections with scale bar indicating 20 nm. The contrast observed in the interface between the Ge nanowire and the Si substrate is due to interdiffusion. (e) and (f) presents the BF TEM image of the cross-section parallel to the nanowire length. The contrast observed at the interface between the Ge and Si substrate is the moiré pattern. The BF TEM image also shows the presence of occasional threading dislocations in (e) and stacking faults in (f).

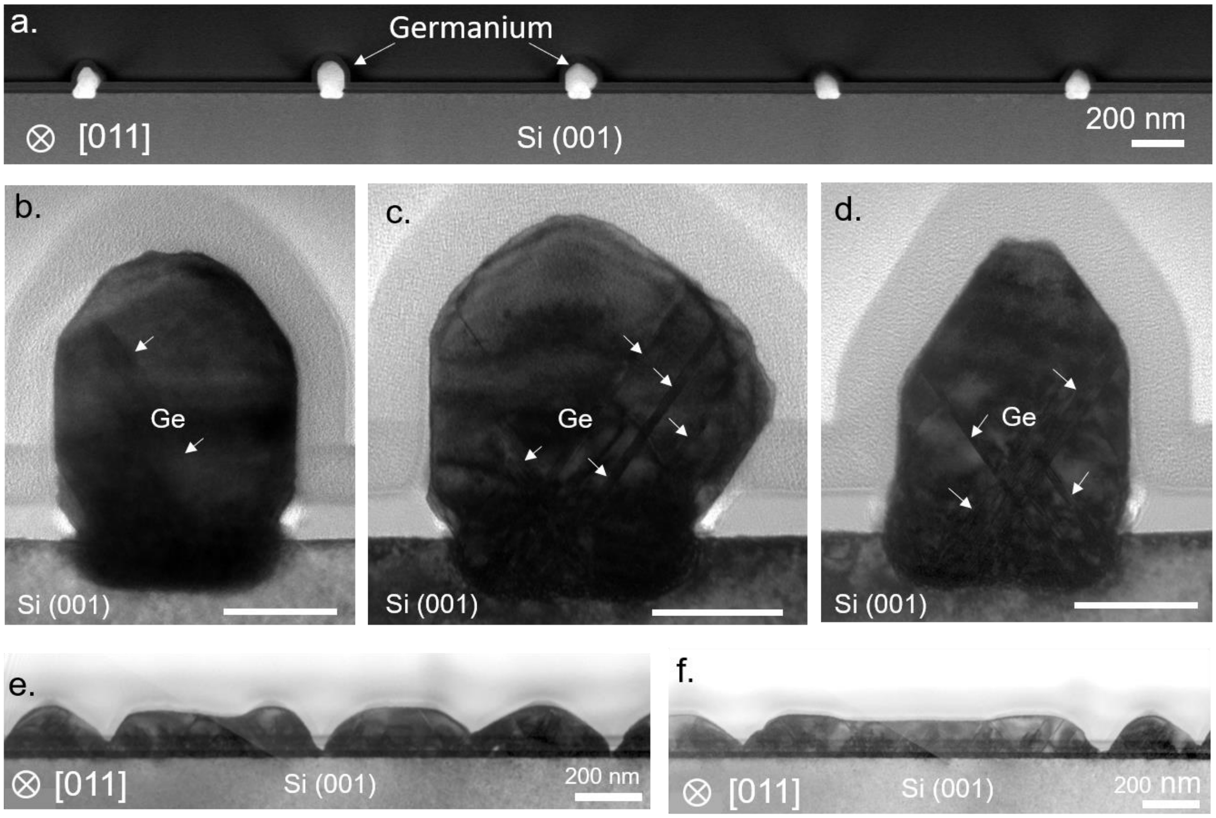

Figure S6: Transmission electron microscopy image of the Ge nanowires grown without AsH_3 during the surface pretreatment step. (a) HAADF STEM image of the Ge nanowire cross-section providing an overview of the cross-sectional morphology. (b-d) BF TEM image of three nanowire cross sections with scale bar indicating 50 nm. The BF TEM images show the presence of a high density of planar defects (indicated by white arrows) and non-uniformity in the cross-sectional morphology. (e) and (f) presents the BF TEM image of the cross-section parallel to the nanowire length. The Ge islands are discontinuous and exhibit a very high density of planar defects.

6. Chemical composition analysis

High angle annular dark field (HAADF) STEM image and EDX elemental maps were collected using FEI Talos transmission electron microscope operating at 200 kv.

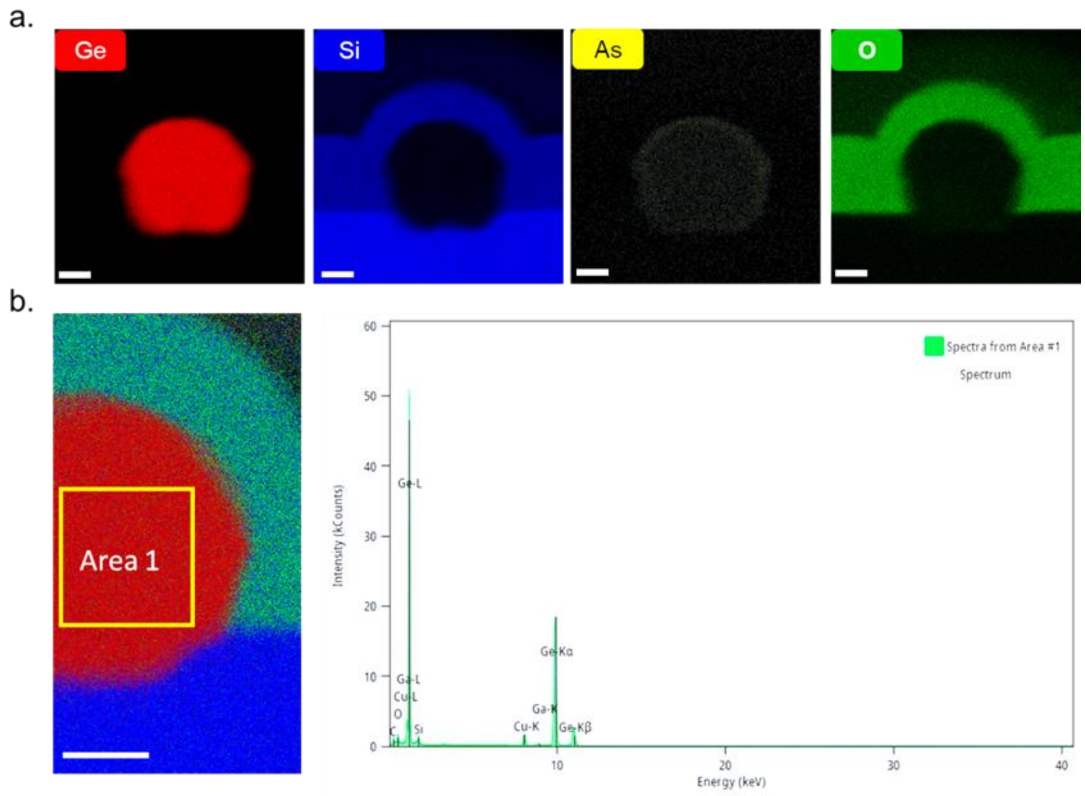

Figure S7: Compositional assessment of the Ge nanowire obtained after surface pretreatment with AsH_3. (a) STEM-EDX elemental mapping showing the distribution of Ge, Si, As, and O, in the Ge nanowire presented in Figure 4. (b) EDX spectra of the Ge nanowire acquired from the area 1. All the presented scalebars indicates 20 nm.

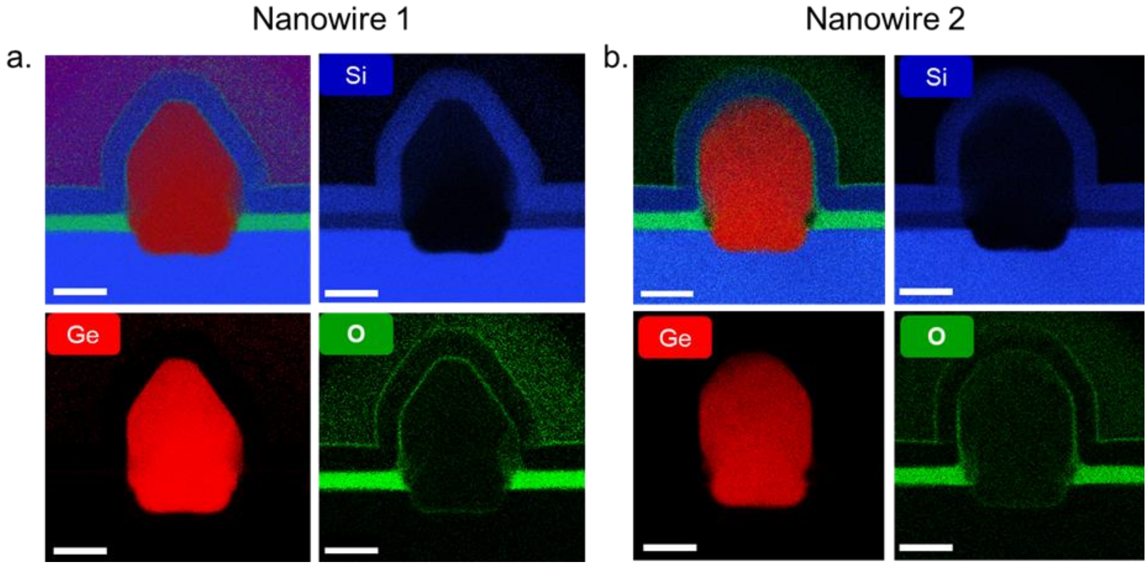

Figure S8: Compositional assessment of the Ge nanowire obtained after surface pretreatment without AsH_3. (a) STEM-EDX elemental mapping showing the distribution of Ge, Si, As and O, in the Ge nanowire presented in Figure 4. (b) STEM-EDX elemental mapping showing the distribution of Ge, Si, As, and O, in an additional nanowire not presented in the main text. Both the nanowire sample shows the presence of an interfacial oxide layer. The scale bar indicates 50 nm.

7. Electrical characterization of the nanowires

We examined the electrical characteristics of the continuous Ge nanowires through Hall measurements, following the procedure outlined in reference [1]. Continuous Ge nanowires were grown in the form of a nanowire network and fabricated into Hall bar devices. In Figure S9, we show a scanning electron microscopy (SEM) image of such a nanowire Hall bar. Ohmic contacts were made using a stack of Ti (10 nm) /Al (30 nm) /Ti (20 nm) /Au (100 nm).

For the top gate fabrication, we initially covered the nanowire Hall bar device with a 30 nm AlO_x atomic layer deposition (ALD) layer, followed by the deposition of metal layers consisting of 10 nm Ti and 90 nm Au. We subsequently conducted Hall measurements on these devices employing standard low-frequency lock-in techniques. The measurements were performed in a ^{4}He cryostat with a base temperature of 1.8 K.

From measurements of the Hall (R_{xy}) and longitudinal (R_{xx}) resistance as a function of perpendicular magnetic field (B) and gate voltage (V_G), we extracted the density and mobility as a function of V_G (Figure S9b). Due to the unknown transport cross section, assumptions about the effective height (H_{eff}) and width (W_{eff}) of the nanowire have been made to extract the hole density and mobility. The plots indicate a large hole density of $1* 10^{18}$ and a mobility of 400 cm^2/Vs at 1.8 K, similar to the values obtained in the previous work. In Figure S9 c and d, we additionally present magneto-transport-measurements at

varying temperatures from 1.8 K to 20 K. These show a clear weak anti-localization peak as well as universal conductance fluctuations, indicating coherent transport over a length scale of the order of L_{xx}.

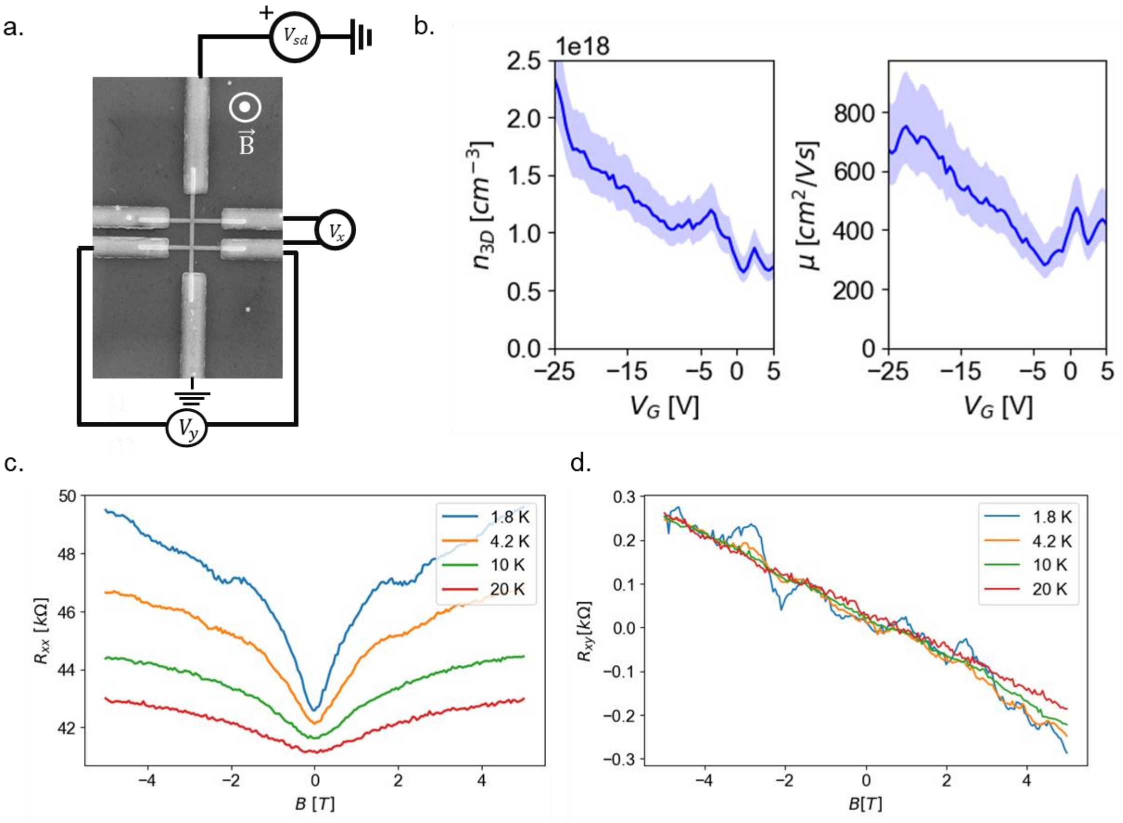

Figure S9: Electrical properties of the Ge nanowires obtained after surface pretreatment with AsH_3. (a) SEM image of the nanowire Hall bar device (b) Hole density (n_{3D} (cm^{-3})) and hall mobility (μ (cm^2/Vs)) as a function of the top gate voltage (V_G (V)). The values are obtained assuming an effective nanowire height (H_{eff}) of 60 ± 10 nm and an effective nanowire width (W_{eff}) of 50 ± 10 nm. The blue shaded regions indicate the uncertainty corresponding to ± 10 nm variations of H_{eff} and W_{eff}.. (c) and (d) respectively show longitudinal (R_{xx}) and Hall (R_{xy}) resistances as a function of perpendicular magnetic field obtained from magneto-transport measurements on an ungated nanowire Hall bar.

8. Atomic resolution HAADF-STEM imaging

Atomic resolution high angle annular dark field scanning TEM (HAADF -STEM) imaging was performed to probe the strain distribution and interface quality between the Ge nanowire and the Si substrate. Figure 6 summarizes the strain distribution and the interface quality of the Ge nanowire obtained after the surface pretreatment step with AsH_3. A summary of the strain distribution and interface quality of the Ge nanowires obtained without AsH_3 during the surface pretreatment step is shown in Figure 7.

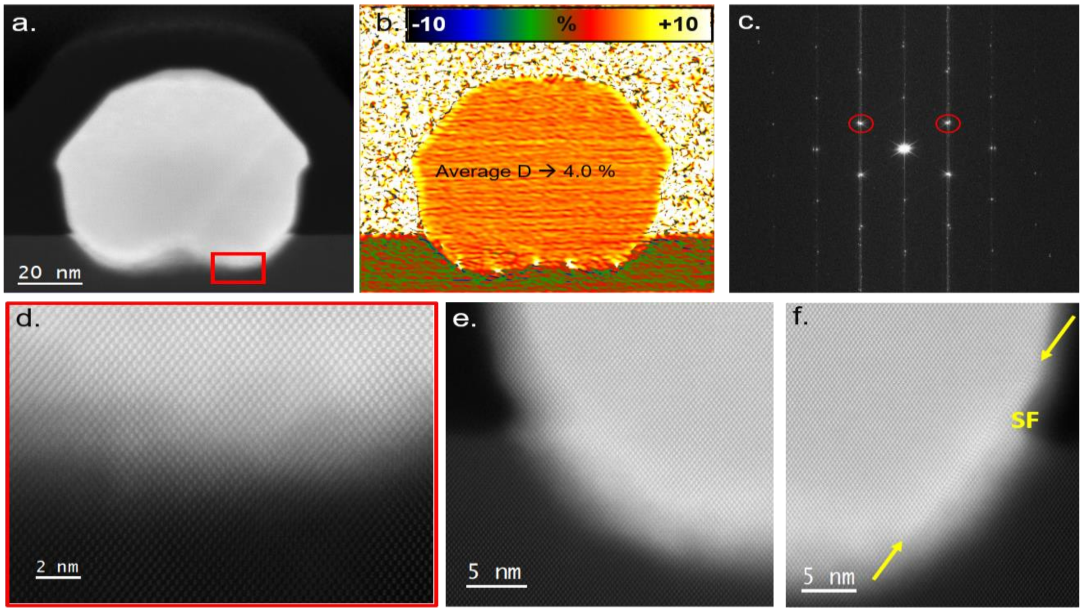

Figure S10: (a) Atomic-resolution HAADF-STEM micrograph showing the nanowire cross-section and faceting. (b) GPA dilation map obtained in the {111} planes showing the presence of misfit dislocations at the Ge-Si interface. The Ge nanostructures are fully relaxed, and GPA analysis gave an average dilation of 4% with respect to the Si substrate. (c) Fast Fourier transform of the HAADF image and the red circle denotes the planes used for GPA. (d - f) Magnified images of the germanium nanowire Si substrate interface. The presence of a diffused interface is an indication of the alloying at the interface.

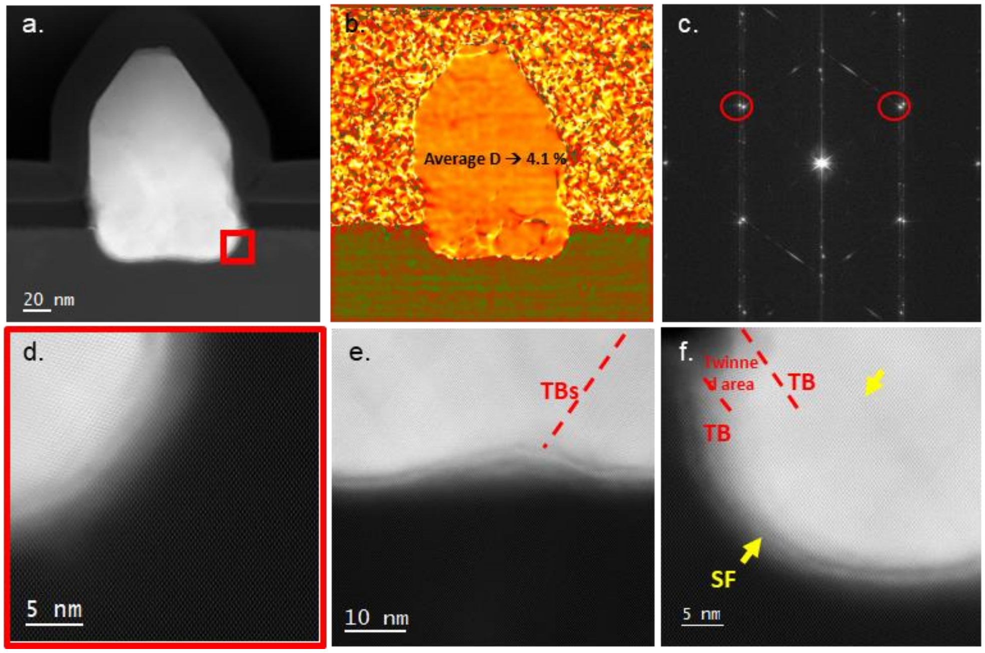

Figure S 11: a) Atomic-resolution HAADF-STEM micrograph showing the nanowire cross-section and faceting. (b) GPA dilation map obtained in the {111} planes showing the presence of misfit dislocations at the Ge-Si interface. The Ge nanostructures are fully relaxed, and GPA analysis gave an average dilation of 4% with respect to the Si substrate. (c) Fast Fourier transform of the HAADF image and the red circle denotes the planes used for GPA. (d - f) The magnified images of the germanium nanowire Si substrate interface show an amorphous interfacial layer.

References:

1. Ramanandan, S. P. *et al.* Coherent Hole Transport in Selective Area Grown Ge Nanowire Networks. *Nano Lett.* **22**, 4269–4275 (2022).
